# Analysis and Correction of Measurement Error of Spherical Capacitive Sensor Caused by Assembly Error of the Inner Frame in the Aeronautical Optoelectronic Pod

**DOI:** 10.3390/s22239543

**Published:** 2022-12-06

**Authors:** Tianxiang Ma, Shengqi Yang, Yongsen Xu, Dachuan Liu, Jinghua Hou, Yunqing Liu

**Affiliations:** 1 Information and Communication Engineering, School of Electronic and Information Engineering, Changchun University of Science and Technology, Changchun 130022, China; 2 Key Laboratory of Airborne Optical Imaging and Measurement, Changchun Institute of Optics, Fine Mechanics and Physics, Chinese Academy of Sciences, Changchun 130033, China; 3 University of Chinese Academy of Sciences, Beijing 100049, China; 4 Jiuquan Satellite Launch Centre, Jiuquan 732750, China

**Keywords:** spherical capacitive angular sensor, 2-DOF angular displacement measurement, four-quadrant differential structure, installation error compensation

## Abstract

The ball joint is a multi-degree-of-freedom transmission pair, if it can replace the inner frame in the aviation photoelectric pod to carry the optical load, which will greatly simplify the system structure of the photoelectric pod and reduce the space occupied by the inner frame. However, installation errors in ball joint siting introduce nonlinear errors that are difficult to correct and two degree of freedom angular displacement of the ball joint is difficult to detect, which limits application in the precision control of two degrees of freedom systems. Studies of spherical capacitive sensors to date have not tested sensors for use in an inner frame stabilisation mechanism nor have they analysed the influence of installation error on sensor output. A two-axis angular experimental device was designed to measure the performance of a ball joint capacitive sensor in a frame stabilisation mechanism in an aeronautical optoelectronic pod, and a mathematical model to compensate for ball joint capacitive sensor installation error was created and tested. The experimental results show that the resolution of the capacitive sensor was 0.02° in the operating range ±4°, the repeatability factor was 0.86%, and the pulse response time was 39 μs. The designed capacitive sensor has a simple structure, high measurement accuracy, and strong robustness, and it can be integrated into ball joint applications in the frames of aeronautical photoelectric pods.

## 1. Introduction

A ball joint has significant technical advantages over a universal joint as a transmission mechanism, such as smaller size, several degrees of freedom of movement, and a shorter transmission path. However, in practical applications, the detection of 2-DOF angular displacement signals of spherical motion pairs has the following problems: coupled 2-DOF angular displacement signals are difficult to decouple, and electrode installation errors introduce nonlinear errors that are difficult to compensate. These problems limit the application of spherical motion pairs in aeronautical photoelectric pods and in other precision equipment, such as robot ball joints and aeronautical optoelectronic pods [1]. Several methods have been devised to detect the 2-DOF angular displacement of a ball joint. Stein et al. [2] designed a spherical angular displacement measurement encoder based on random Thiessen polygons. The spherical motor rotor was coloured in black and white to encode information of the angular position in binary form. The encoder detected the binary coding of the surface of the ball joint through a photoelectric sensor and calculated the 2-DOF angular displacement. Although this method detected the 2-DOF angular displacement of the ball joint in real time, such methods have the following problems: the optical sensor is very susceptible to environmental interference; the resolution of angle detection is heavily dependent on the fineness of image feature etching; and the detection system is optically complex and voluminous, making it unsuitable for application in compact, lightweight or small devices. To compensate for the shortcomings of optical measurement methods, research has been directed toward methods to detect angular motion that use electromagnetic signals. Foong et al. [3] proposed a multi-dimensional angle detection device based on the magnetic field of a single-layer permanent magnet. The permanent magnet was arranged around the rotor to form a spherical array. It used multi-axis sensors such as Hall sensors or giant magnetoresistive sensors to detect changes in the magnetic field of the permanent magnet array when the joint rotated to determine the angle between the two degrees of freedom. This method overcomes the shortcomings inherent in optical detection, but it still has the following problems: the spherical motion pair can easily rotate out of the sensitive area of the magnetic field, and the magnetic field fluctuates only within a small range, which affects the accuracy of angle detection. Eddy current sensors, in contrast to magnetic field sensors, have the advantages of highly accurate measurement, sensitive responses, and a high capacity to resist interference. Their unique sensing principles promote their wide use in applications that require noncontact measurement. Hu et al. [4] proposed an angle detection method using eddy current sensors. In this method, unique shapes were etched into the surface of the ball head and several eddy current sensors were incorporated into the ball socket to form a sensor array. When the spherical motion pair rotated, the shapes on the metal surface moved and appeared to change. The eddy current sensors detected changes in the intensity of induced eddy currents on the metal surface, and the data they produced were used to calculate the 2-DOF angular displacement of the spherical joint. However, this approach is highly demanding on the sensor location and the positioning of the eddy current sensor array. It is easy to introduce superposition errors, and the processing required is computation-intensive. Measurement accuracy and resolution are easily affected by the machining accuracy of the surface shape features of the ball joints. A capacitive sensor has the advantages of high accuracy, low cost, and simple structure. These features make it suitable for use in applications where precise measurements are required, such as spherical pairs, CNC machine tools, and noncontact force–distance measurement [5,6,7,8]. These advantages over other sensing methods have ensured that the capacitive angle sensor has been researched in great depth [9]. This type of capacitive sensor detects 2-DOF angular displacement by calculating change in capacitance due to joint rotation. The sensor has the advantages of being compact and measuring with a high degree of accuracy, both of which make it suitable for measuring the 2-DOF angular displacement of the joint. However, the capacitive sensor does not take account of the effect of electrode installation errors on output signals. Wang et al. [10,11] analysed the influence of electrode mounting error on measurement signals, but they did not investigate the effects of installation error on 2-DOF angular displacement. In this study, we mathematically modelled the installation error of the sensor in practical applications and verified the model using finite element analysis. We then designed and conducted a two-axis experiment to test the static characteristics, dynamic characteristics, and other properties of the capacitive sensor. The experimental results show that the measurement errors introduced by mounting errors are greatly reduced, which shows the sensor has good robustness, can meet the detection needs of the angle signal for two degrees of freedom, and is suitable for installation in the frame of an aeronautical photoelectric pod.

## 2. Measurement Principle

We propose a capacitive sensor with a spherical pair structure that operates on the principle of variable area capacitive measurement. We infer the functional relationship between the 2-DOF angular displacement signal of the spherical capacitive sensor and the theoretical output capacitance of the spherical capacitive sensor. We then propose a decoupling and calculation method for the 2-DOF angular displacement signal derived from analysis of the mathematical model that we created.

### 2.1. Structural Model of the Spherical Capacitive Sensor

**Figure 1:**
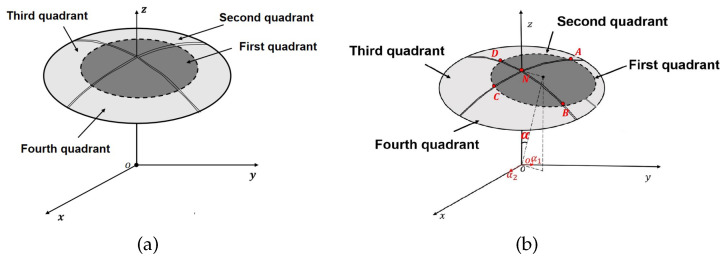
The sensor structure schematic diagram: (**a**) the four-quadrant differential capacitance is balanced; (**b**) the four-quadrant differential capacitance is unbalanced.

Figure 1 shows that when the sensing electrode is in the initial position, the area between the two electrodes is equal. In that condition, the four-quadrant differential capacitance is balanced, and the sensor output is null. When the sensing electrode rotates through a spatial angle, the area of overlap between the two electrodes changes, causing an imbalance between the four-quadrant differential capacitance, resulting in a difference in the capacitance of the four measurement capacitors, and then the sensor outputs the differential capacitance signals $\Delta C_{13}$ and $\Delta C_{24}$. This difference can be quantified by detecting the capacitances $\Delta C_{13}$ and $\Delta C_{24}$, computing the angle components of the sensor around the x-axis $\alpha_{1}$ and the y-axis $\alpha_{2}$.

### 2.2. 2-DOF Angular Displacement Signal Measurement

Ignoring the edge effect, the differential output capacitances $C_{13}$ and $C_{24}$ of the sensor can be written as a matrix λ($\frac{\epsilon HR}{rd}=t$) [9]:

(1) $$\lambda =\left[\begin{array}{cccc} t\left(\alpha_{1}+\alpha_{2}\right) & t\left(\alpha_{1}-\alpha_{2}\right) & -t\left(\alpha_{1}+\alpha_{2}\right) & -t\left(\alpha_{1}-\alpha_{2}\right)\\ t\left(\alpha_{1}-\alpha_{2}\right) & t\left(\alpha_{1}+\alpha_{2}\right) & -t\left(\alpha_{1}-\alpha_{2}\right) & -t\left(\alpha_{1}+\alpha_{2}\right)\\ -t\left(\alpha_{1}+\alpha_{2}\right) & -t\left(\alpha_{1}-\alpha_{2}\right) & t\left(\alpha_{1}+\alpha_{2}\right) & t\left(\alpha_{1}-\alpha_{2}\right)\\ -t\left(\alpha_{1}-\alpha_{2}\right) & -t\left(\alpha_{1}+\alpha_{2}\right) & t\left(\alpha_{1}-\alpha_{2}\right) & t\left(\alpha_{1}+\alpha_{2}\right) \end{array}\right].$$

where ϵ is the air dielectric constant, and *H* is $-Rr(\pi -2arccos\frac{r}{R})$.

The voltage excitation matrix δ($\omega_{1}\neq \omega_{2}$) is:

(2) $$\delta =\left[\begin{array}{c} A\sin\left(\omega_{1}t\right)+A\sin\left(\omega_{2}t\right)\\ A\sin\left(\omega_{1}t\right)-A\sin\left(\omega_{2}t\right)\\ -A\sin\left(\omega_{1}t\right)-A\sin\left(\omega_{2}t\right)\\ -A\sin\left(\omega_{1}t\right)+A\sin\left(\omega_{2}t\right) \end{array}\right]$$

where *A* is the amplitude of the two sinusoidal excitation signals, and $\omega_{1}t$ and $\omega_{2}t$ are the angular frequency of two sinusoidal excitation signals.

After the voltage excitation decoupling operation λ·δ, the 2-DOF angular displacement signals coupled in the differential output capacitor are converted into voltage signals with different frequencies.

(3) $$V_{Sig}=\left[\begin{array}{c} V_{1}\sin\left(\omega_{1}t\right)+V_{2}\sin\left(\omega_{2}t\right)\\ V_{1}\sin\left(\omega_{1}t\right)-V_{2}\sin\left(\omega_{2}t\right)\\ -V_{1}\sin\left(\omega_{1}t\right)-V_{2}\sin\left(\omega_{2}t\right)\\ -V_{1}\sin\left(\omega_{1}t\right)+V_{2}\sin\left(\omega_{2}t\right) \end{array}\right].$$

$V_{1}=4AG\alpha_{1}\omega j$, $V_{2}=4AG\alpha_{2}\omega j$, and G is the gain of the transconductance amplifier circuit. The i‒th row of $V_{Sig}$ represents the voltage signal output by the capacitive sensor when the sensing electrode enters the i‒th quadrant (i=1,2,3,4). We can caculate $\alpha_{1}$ and $\alpha_{2}$ by demodulating $V_{Sig}$.

### 2.3. Calculation of Electrode Installation Error

**Figure 2:**
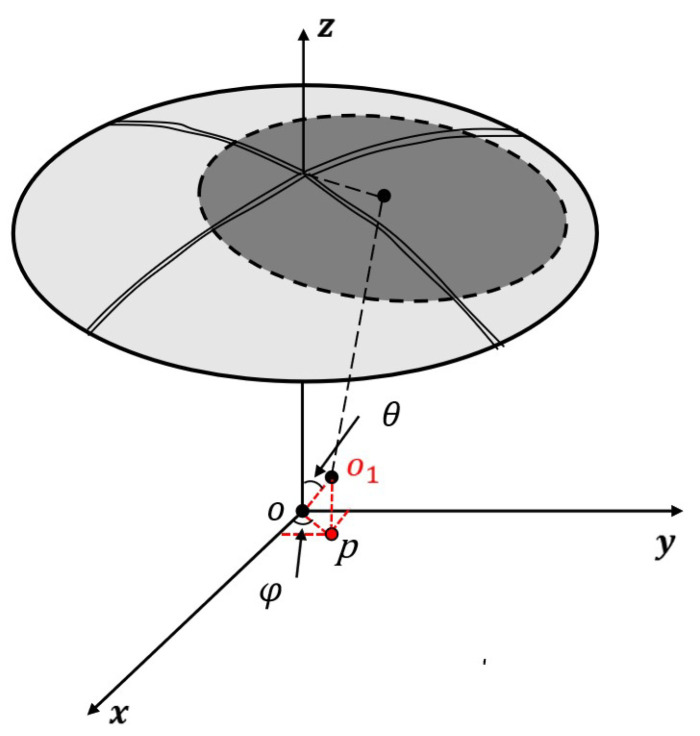
Functional block diagram.

The structural parts that fix the spherical electrode will have shape errors during processing. Due to shape errors of the structural parts, the spherical centre of the sensing electrode and the spherical centre of the driving electrode no longer coincide in assembling the electrode, causing the spherical centre of the sensing electrode to have an offset. Figure 2 shows that when the installation error of the two spherical electrodes has a single degree of freedom offset $\delta_{x}$/$\delta_{y}$/$\delta_{z}$ along the *x*-/*y*-/*z*-axis, the parameters ΔA and d will be changed simultaneously. The mounting error δ is a complex function of the differential output capacitance of the sensor. Therefore, modelling the installation error of the spherical capacitive sensor will accurately represent the influence of installation error on measurements and so will greatly improve the measurement accuracy of the capacitive sensor.

The mathematical relationship between the electrode gap d and the mounting error is [11]:

(4) $$d=R-r-\lambda_{x}sin\left(\theta \right)cos\left(\phi \right)-\lambda_{y}sin\left(\theta \right)sin\left(\phi \right)-\lambda_{z}cos\left(\theta \right)$$

and the differential output capacitance of the spherical capacitive sensor is therefore calculated as:

(5) $$\Delta C_{13}=\underset{ANB}{\;\int \int \;}\frac{\epsilon }{d}d\sigma -\underset{DNC}{\;\int \int \;}\frac{\epsilon }{d}d\sigma$$

(6) $$\begin{array}{c} \Delta C_{24}=\underset{DNA}{\;\int \int \;}\frac{\epsilon }{d}d\sigma -\underset{BNC}{\;\int \int \;}\frac{\epsilon }{d}d\sigma \end{array}$$

When the centre of the sensing electrode shifts into the first quadrant, and the sensing electrode moves from the third quadrant to the first quadrant, after integration and simplification, the differential capacitance expression of the capacitive sensor is:

(7) $$\Delta C_{13}=\frac{\epsilon Rr(\pi -2arccos\left(\frac{r}{R}\right))}{d+\lambda_{z}}\left(\pi Rarcsin\left(\frac{\lambda_{x}}{R}\right)+R\alpha_{1}+\pi Rarcsin\left(\frac{\lambda_{y}}{R}\right)+R\alpha_{2}\right)$$

(8) $$\Delta C_{24}=\frac{\epsilon Rr(\pi -2arccos\left(\frac{r}{R}\right))}{d+\lambda_{z}}\left(\pi Rarcsin\left(\frac{\lambda_{x}}{R}\right)+R\alpha_{1}+\pi Rarcsin\left(\frac{\lambda_{y}}{R}\right)-R\alpha_{2}\right)$$

When the centre of the sensing electrode shifts into the first quadrant, and the sensing electrode moves from the fourth quadrant to the second quadrant, after integration and simplification, the differential capacitance expression of the capacitive sensor is:

(9) $$\Delta C_{13}=\frac{\epsilon Rr(\pi -2arccos\left(\frac{r}{R}\right))}{d+\lambda_{z}}\left(-\pi Rarcsin\left(\frac{\lambda_{x}}{R}\right)+R\alpha_{1}-\pi Rarcsin\left(\frac{\lambda_{y}}{R}\right)+R\alpha_{2}\right)$$

(10) $$\Delta C_{24}=\frac{\epsilon Rr(\pi -2arccos\left(\frac{r}{R}\right))}{d+\lambda_{z}}\left(-\pi Rarcsin\left(\frac{\lambda_{x}}{R}\right)+R\alpha_{1}-\pi Rarcsin\left(\frac{\lambda_{y}}{R}\right)-R\alpha_{2}\right)$$

Based on the decoupling operation λ•δ, we can obtain the decoupling output expression with the installation error:

(11) $$\begin{array}{cc} V_{1} & =\frac{2\epsilon Rr(\pi -2arccos\left(\frac{r}{R}\right))}{d+\lambda_{z}}\left(-2\pi Rarcsin\left(\frac{\lambda_{x}}{R}\right)-2\pi Rarcsin\left(\frac{\lambda_{y}}{R}\right)+2R\alpha_{1}\right)G\omega_{1}sin\left(\omega_{1}t\right) \end{array}$$

(12) $$V_{2}=\frac{2\epsilon Rr(\pi -2arccos\left(\frac{r}{R}\right))}{d+\lambda_{z}}\left(2R\alpha_{2}\right)G\omega_{2}sin\left(\omega_{2}t\right)$$

## 3. Finite Element Model Setup

We built the 3D finite element simulation model by Ansoft Maxwell software to verify Equations (11) and (12). The effect of the installation error on sensor output was determined by comparing the results of the finite element model with the installation error of the mathematical model [12,13].

### 3.1. Model Parameters for the Capacitive Sensor

**Figure 3:**
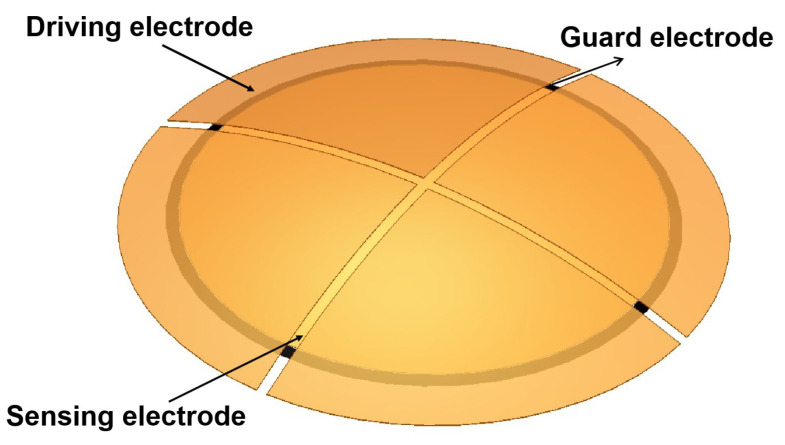
Model of the capacitive sensor.

The sensor’s parameters are listed in Table 1 [9], and the 3D finite element simulation model is shown in Figure 3.

When assembling the frame, the minimum thickness of the standard gasket for adjusting the mounting position of the frame is 0.01 mm. Therefore, finite element models were created with installation errors of 5 μm (5 μm < 0.01 mm) and 50 μm (50 μm > 0.01 mm) to determine the effect of electrode mounting errors on capacitive sensor output. Comparison between the finite element model and the mathematical models allowed us to determine the influence of installation error on the capacitive sensor output curves.

We moved the sensing electrode in the directions of $30^{\circ }$ and $45^{\circ }$ and determined the effects of the installation errors $\left(\delta_{x},\delta_{y},0\right)$, $\left(0,0,\delta_{z}\right)$, and $\left(\delta_{x},\delta_{y},\delta_{z}\right)$ on the capacitive sensor output curve. In the model, the range of the 2-DOF angular displacement was set to [−4${}^{\circ }$, 4${}^{\circ }$], and the step was $0.1^{\circ }$. $\Delta C_{13}\_without$ and $\Delta C_{24}\_without$ represent the differential output capacitance of the mathematical model without installation errors, and $\Delta C_{13}\_with$ and $\Delta C_{24}\_with$ are the differential output capacitance of the mathematical model with installation errors. $\Delta C_{13}\_finite$ and $\Delta C_{24}\_finite$ represent the predicted differential output capacitances of the finite element model.

**Figure 4:**
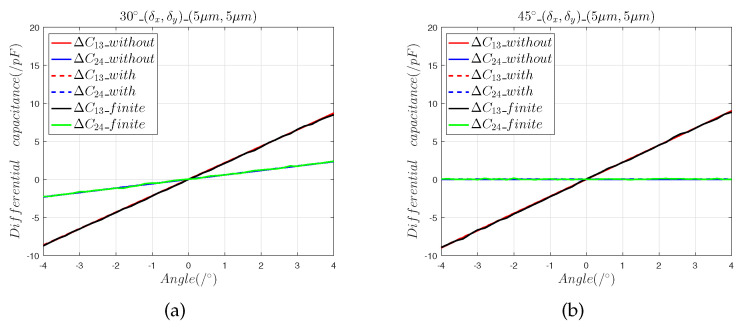
The 5 μm axial installation error analysis: (**a**) differential capacitance in the $30^{\circ }$ direction; (**b**) output capacitance in the $45^{\circ }$ direction.

**Figure 5:**
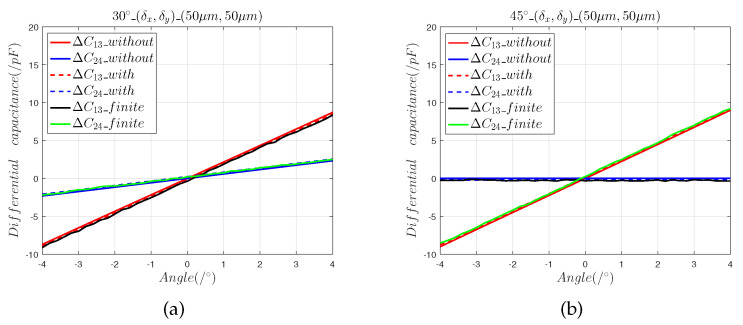
The 50 μm axial installation error analysis: (**a**) differential capacitance in the 30° direction; (**b**) output capacitance in the 45° direction.

Figure 4 and Figure 5 show that:

(1) The results of the finite element model are consistent with the predictions calculated using Equations (7) and (8), which shows that the capacitance calculation model with an installation error can accurately predict the effects of the axial installation error on angle measurement.
(2) The 5 μm axial installation error introduced a zero offset error of 0.012° and the 50 μm axial installation error introduced a zero offset error of 0.11°.
(3) When the sensing electrode moves along the space angles $30^{\circ }$ and $45^{\circ }$, the 5 μm axial installation errors and the 50 μm axial installation errors introduce equal zero offset errors, which indicates that the axial installation error can be reduced by calibration.

**Figure 6:**
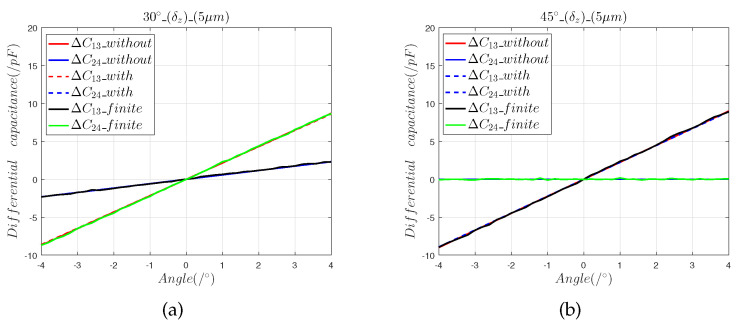
The 5 μm radial installation error analysis: (a) differential capacitance in the 30° direction; (b) output capacitance in the 45° direction.

**Figure 7:**
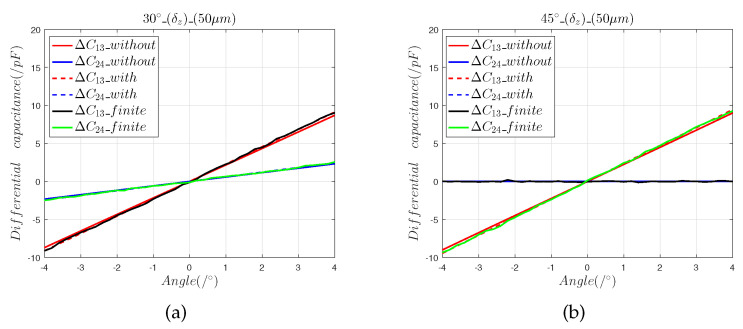
The 50 μm radial installation error analysis: (**a**) differential capacitance in the 30° direction; (**b**) output capacitance in the 45° direction.

Figure 6 and Figure 7 show that:

(1) The results of the finite element model that contained the radial installation error are consistent with the predictions of the mathematical model. The radial installation error will cause the measurement results of the sensor to have a slope offset, which is defined as:
  (13) $$\eta =\frac{\left|S_{fitted}-S_{theoretical}\right|}{S_{theoretical}}\times 100\%$$
(2) Radial installation errors differ from axial installation errors in that they cause the output curve of the sensor to produce a slope offset error. It can be seen that in the 30° direction, the 5 μm radial installation error introduced a 0.7% slope offset error, and the 50 μm radial installation error introduced a 4.79% slope offset error. In the 45° direction, the 5 μm radial installation error introduced a slope offset error of 0.8%, and the 50 μm radial installation error introduced a 4.83% slope offset error.

**Figure 8:**
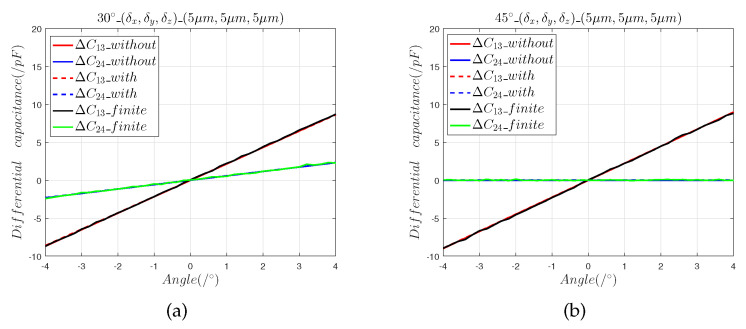
The 5 μm composite installation error analysis: (**a**) differential capacitance in the 30° direction; (**b**) differential capacitance in the 45° direction.

**Figure 9:**
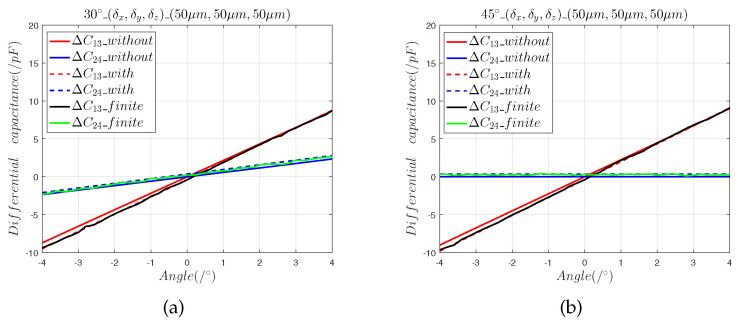
The 50 μm composite installation error analysis: (**a**) differential capacitance in the 30° direction; (**b**) differential capacitance in the 45° direction.

Figure 8 and Figure 9 show that:

(1) The results calculated by the finite element model are consistent with the predictions of the mathematical model, which shows that the mathematical model accurately calculated the value of the installation error.
(2) Composite mounting errors cause nonlinear offsets. In the 30° direction, the maximum angle measurement error introduced by the composite installation errors of 5 μm and 50 μm were, respectively, 0.018° and 0.357°; the corresponding values for the 45° direction were 0.0156° and 0.354°

The 3D finite element model was created to verify the mathematical model of Equations (7)–(10). The results given by the 3D finite element model demonstrate that Equations (7)–(10) accurately predict the effects of installation error.

### 3.2. Modelling and Analysis of Installation Error Correction Methods

In this section, we establish a correction model to correct measurement errors introduced by the installation errors, based on Equations (7)–(10), and verify the correction model by comparison with the finite element model.

**Figure 10:**
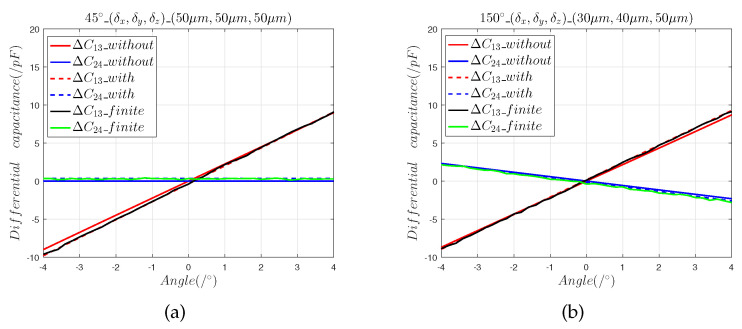
The (30 μm, 40 μm, and 50 μm) composite installation errors analysis: (**a**) differential capacitance in direction $45^{\circ }$; (**b**) differential capacitance in direction $150^{\circ }$.

To visually see the compensation effect of the compensation method on the measurement error introduced by the installation error, we take the irregular installation errors (30 μm, 40 μm, and 50 μm) ($\lambda_{x}\neq \lambda_{y}\neq \lambda_{z}$) greater than 0.01 mm as examples to analyse the compensation method for correcting installation errors. The analysis results are shown in Figure 10.

**Figure 11:**
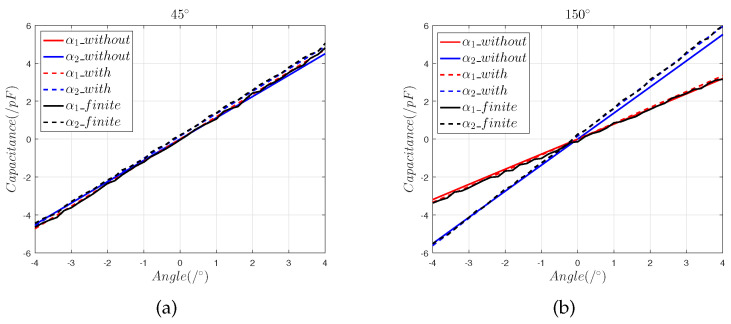
The (30 μm, 40 μm, and 50 μm) composite installation error analysis: (**a**) differential capacitance in the 45° direction; (**b**) differential capacitance in the 150° direction.

By solving Equations (11) and (12), we obtain the decoupled angular displacement signals $\alpha_{1}\_with$ and $\alpha_{2}\_with$ that include installation error. Figure 11 shows the finite element model results $\alpha_{1}\_finite$ and $\alpha_{2}\_finite$; the model predictions including installation errors, $\alpha_{1}\_with$ and $\alpha_{2}\_with$; and the model predictions without installation errors, $\alpha_{1}\_without$ and $\alpha_{2}\_without$.

**Figure 12:**
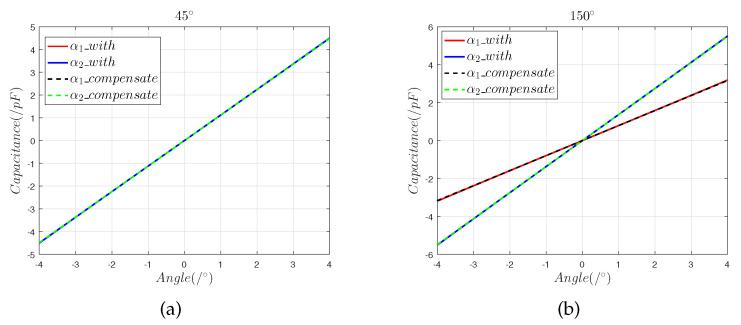
The 50 μm composite installation error analysis: (**a**) differential capacitance in the 45° direction; (**b**) differential capacitance in the 150° direction.

Figure 11 shows that:

(1) The curve $\alpha_{2}$ only has a slope offset, which is consistent with Equation (12). According to the slope of the $\alpha_{2}$ fitting curve and the slope of the theoretical curve, the offset $\lambda_{z}$ can be obtained.
(2) Since the effects of the slope offset error introduced by the offset $\lambda_{z}$ differ for different trajectories, this part of the error cannot be eliminated by calibration, so $\lambda_{z}$ can be compensated for by multiplying by $\frac{\lambda_{z}+d}{d}$.
(3) The zero offset error introduced by the offsets $\lambda_{x}$ and $\lambda_{y}$ is a fixed value on different trajectories. We solve the expression to obtain the zero offset error introduced by $\lambda_{x}$+$\lambda_{y}$ and subtract the solution value from the error value to correct for $\lambda_{x}$+$\lambda_{y}$.
(4) The decoupled output values for installation errors (30 μm, 40 μm, and 50 μm) after correction are shown in Figure 12

Figure 12 shows that the results of the sensor model that include installation error after correction are consistent with the results of the ideal sensor model, which demonstrates that the correction method is accurate.

## 4. Experimental Setup

We designed a spherical capacitive sensor and created a two-axis experiment. Additionally, the processing steps of the capacitive sensors are as follows:

(1) Selecting a material with strong corrosion resistance and low temperature coefficient as the substrate for spherical electrodes; in this study, we select devitrified glass as the substrate material.
(2) The devitrified glass is processed by machining to construct the spherical electrode substrate, and the surface of the processed spherical substrate is precision-ground to make its surface roughness reach the nanometer standard.
(3) The surface of the substrate is coated with gold film, and the details of the electrode, such as terminals and guard ring, can be processed by photolithography.

**Figure 13:**
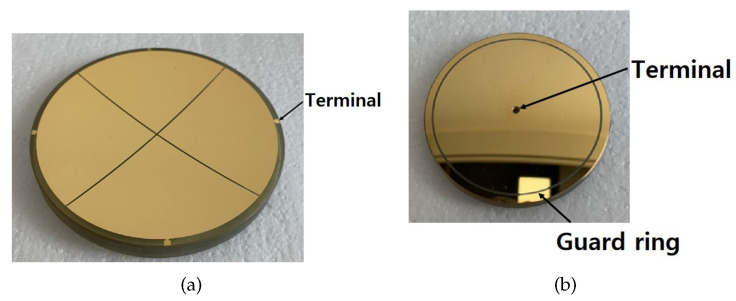
The spherical electrodes design and processing: (**a**) the drive electrode; (**b**) the sense electrode.

The actual electrode is shown in Figure 13, and the dimensions are shown in Table 2.

To test the capability of the designed capacitive sensor to measure the 2-DOF angular displacement signal, we conducted separate single-axis and dual-axis measurement experiments. Data errors, such as the zero offset and slope offset errors in the measurement experiment results, were analysed using the installation error model we created, and we propose a correction method.

### 4.1. Experimental Setup and Assembly

The experimental setup consisted primarily of two spherical electrodes, a 3-DOF linear displacement platform, an encoder, a counterweight block, an adjustable support rod, and a base. In assembling the experimental device, we used the bottom surface of the experimental device as the baseline for positioning the remaining parts. The specific installation process was as follows.

**Figure 14:**
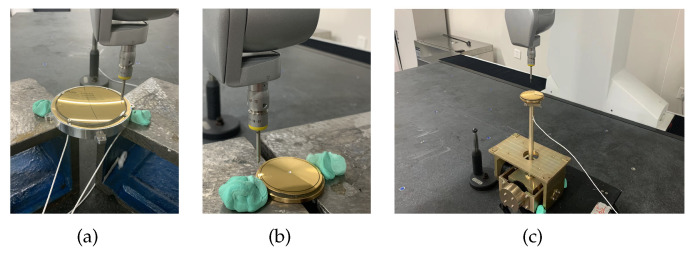
Altitude datum measurement operations: (**a**) $h_{1}$ measurement; (**b**) $h_{2}$ measurement; (**c**) $h_{3}$ measurement.

**Figure 15:**
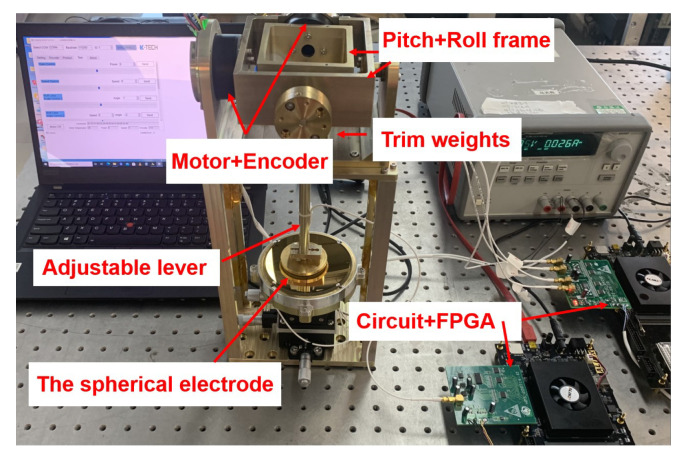
Capacitive sensor system.

(1) The driver electrode was installed, and the sensing electrode was fastened to the brass base. The sensor core wire was attached to the base to prevent it from disengaging from the electrode under external force.
(2) Referring to Figure 14, the height $h_{1}$ of the driving electrode above the brass base, the height $h_{2}$ of the induction electrode above the brass base, and the height $h_{3}$ of the brass base above the bottom surface of the frame were measured by a Croma precision micrometer. The support rod was adjusted according to the measured values of $h_{1}$, $h_{2}$, and $h_{3}$ to reduce radial installation error between the actual position of the induction electrode and the theoretical installation position.
(3) The counterweight flange was adjusted to ensure that the sensing electrode would remain stable at any point in space absent external forces. An image of the physical two-axis experimental frame system is shown in Figure 15.
(4) The capacitances of $C_{1}$, $C_{2}$, $C_{3}$, and $C_{4}$ in the four quadrants were measured with a high-precision LCR meter. According to the measured capacitance values, the x-axis and y-axis of the 3-DOF linear displacement platform were adjusted to preliminarily align the sensing electrode and the driver electrode on the xoy plane. At this time, according to the values of $h_{1}$, $h_{2}$, and $h_{3}$, the installation position of the drive electrode in the z-axis direction was determined.

### 4.2. Signal Processing Circuit

**Figure 16:**
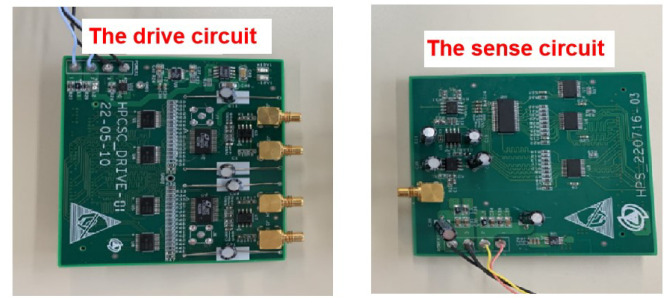
PCB of the signal processing circuit.

The PCB of the signal processing circuit is shown in Figure 16.

**Figure 17:**
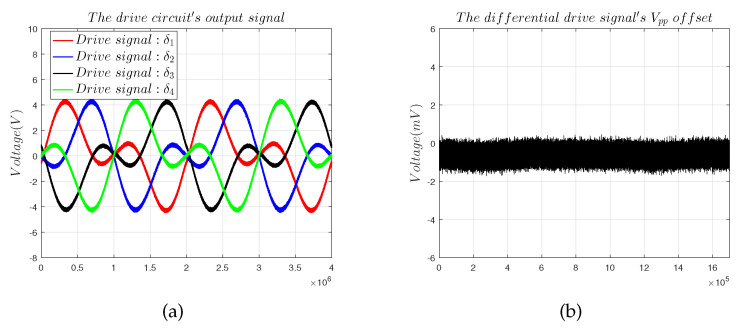
Signal processing circuit output curves: (**a**) driver circuit output curves; (**b**) sensing circuit output curves.

The signal processing circuit consists of a driver circuit and a sensing circuit. The driver circuit outputs the excitation signal matrix δ is shown in Figure 17. Under the excitation of δ, the capacitive sensor outputs a current signal $I_{Sig}$ to realise C/I. The sensing circuit recognises the decoupled angular displacement signal and converts it into the voltage signal matrix $V_{Sig}$ to complete the decoupling operation.

### 4.3. Single-Axis Measurement Experiments

In the single-axis drive measurement experiment, only the pitch shaft motor was driven, so the sensor electrode swings back and forth in the ±5° roll direction from 0°, and the roller shaft remains motionless.

**Figure 18:**
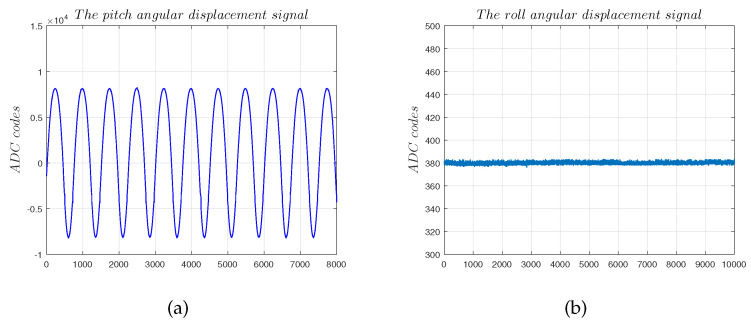
Single-axis measurement experiments: (**a**) solution results of the angular displacement signal $\alpha_{1}$; (**b**) solution results of the angular displacement signal $\alpha_{2}$.

Figure 18 shows that the system’s output matches the oscillation of the motor to output real-time data. However, the system output still has a zero offset of 360 ADC codes that is caused by residual installation errors and the DC component of the circuit.

### 4.4. Dual-Axis Measurement Experiments

This experiment was intended to verify that the spherical capacitive sensor accurately detected the 2-DOF angular displacement signal. During the experiment, the pitch and roll motor was driven to make the sensing electrode move from the third quadrant (4°, −4°) to the first quadrant (4°, 4°) along a 45° trajectory, and the output signal of the spherical capacitive sensor was analysed.

**Figure 19:**
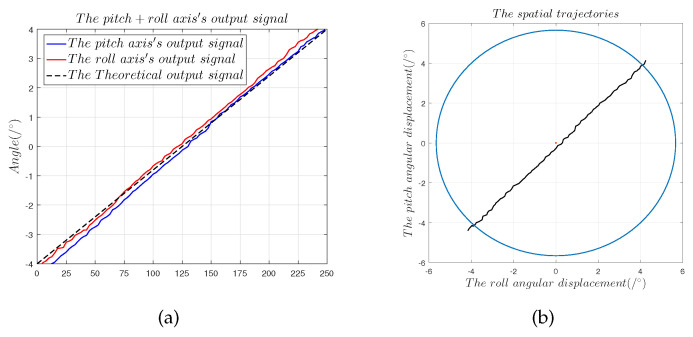
Dual-axis measurement experiments in the 45° direction: (**a**) solution results of the angular displacement signals $\alpha_{1}$ and $\alpha_{2}$; (**b**) 45° displacement trajectories fitted by $\alpha_{1}$ and $\alpha_{2}$.

**Figure 20:**
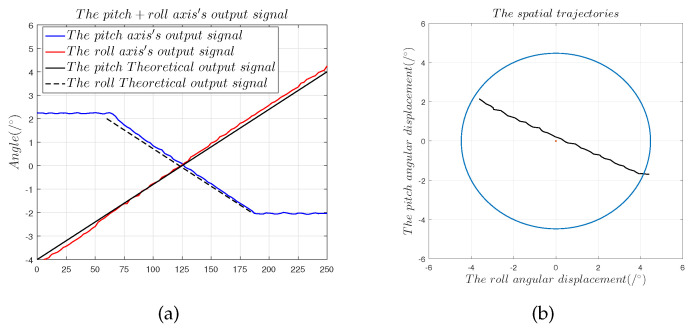
Dual-axis measurement experiments in the 150° direction: (**a**) solution results of the angular displacement signals $\alpha_{1}$ and $\alpha_{2}$; (**b**) 150° displacement trajectories fitted by $\alpha_{1}$ and $\alpha_{2}$.

Figure 19 and Figure 20 show that

(1) The spatial displacement trajectory of the spherical capacitive sensor fitted by $\alpha_{1}$ and $\alpha_{2}$ conformed to the motor drive trajectory, which shows that the designed spherical capacitive sensor correctly detected the 2-DOF angular motion signal when the sensing electrode moved within quadrants 1–4.
(2) The maximum measurement error in the 45° direction was 0.42°, the nonlinearity error was ±5.25%, the zero offset error was 0.2°, and the slope offset error was 9.17%, which translate to $\lambda_{z}$ = 14.4 μm.
(3) The maximum measurement error in the 150° direction was 0.36°, the nonlinearity error was ±4.5%, the zero offset error was 0.198°, and the slope offset error was 6.75%, which translate to $\lambda_{z}$ = 14.8 μm.
(4) The preceding results show that the capacitive sensor with a four-quadrant differential structure is capable of strong common-mode noise rejection, which indicates that measurement error introduced by common-mode noise, the cable parasitic capacitance $C_{couple}$, and the additional capacitance introduced by edge effects do not cause a slope offset error. Equation (12) shows that only the electrode gap d can induce a slope offset error in the capacitive sensor. Thus, measurement error is mainly governed by installation error [9].

### 4.5. Installation Error Compensation and Correction

A compensation operation was added to the FPGA solution process according to the zero offset error and slope offset error determined. The steps of the compensation operation were first to multiply the solution data by the slope offset compensation operator $\frac{\lambda_{z}+d}{d}$ to correct the measurement error introduced by $\lambda_{z}$, taking $\lambda_{z}$ =14.8 μm. Secondly, the measurement error introduced by $\lambda_{x}$+$\lambda_{y}$ was corrected by subtracting the measured zero offset error ($0.2^{\circ }$) from the solution data.

**Figure 21:**
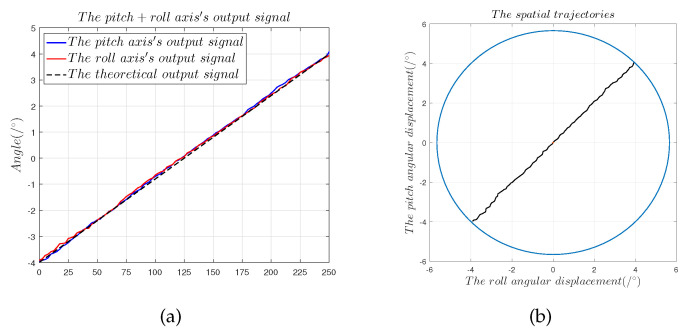
Dual-axis compensation experiments in the 45° direction: (**a**) compensation results of the angular displacement signals $\alpha_{1}$ and $\alpha_{2}$; (**b**) 45° displacement trajectories fitted by $\alpha_{1}$ and $\alpha_{2}$.

**Figure 22:**
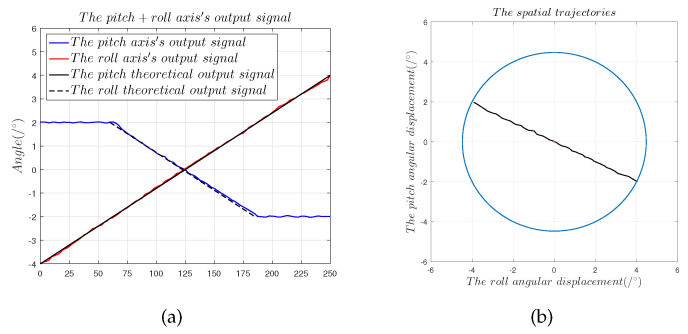
Dual-axis compensation experiments in the 150° direction: (**a**) compensation results of the angular displacement signals $\alpha_{1}$ and $\alpha_{2}$; (**b**) 150° displacement trajectories fitted by $\alpha_{1}$ and $\alpha_{2}$.

Figure 21 and Figure 22 show that after the compensation operation, the slope offset in the 45° direction was reduced from 9.17% to 0.68%, and the nonlinearity error in the 45° direction was reduced to 0.975%. In the 150° direction, the slope offset was reduced from 6.75% to 0.52%, and the nonlinearity error was reduced to 0.837%. In order to verify the stability of the compensation operation, the results in the 45° direction were used as an example to determine the hysteresis error, the repeatability error, the sensitivity, and the resolution of the capacitive sensor.

#### 4.5.1. Hysteresis Error and Repeatability Error

The sensor hysteresis error is the degree to which the output characteristics of the sensor do not coincide in forward and backward motion. The hysteresis error is given by:

(14) $$e_{t}=\frac{\Delta U_{Max}}{U_{Fs}}\times 100\%$$

where $e_{t}$ is the hysteresis error, and $\Delta U_{Max}$ is the greatest deviation in forward and backward movement. Figure 22 shows that when the capacitive sensor moves from $\left(-4^{\circ },-4^{\circ }\right)$ to $\left(4^{\circ },4^{\circ }\right)$, the hysteresis error was 0.93%. The repeatability error is a measure of the extent to which sensor output is consistent for repeated movements in the same direction and range. The repeatability error is given by:

(15) $$e_{z}=\pm \frac{3\sigma }{U_{Fs}}\times 100\%$$

**Figure 23:**
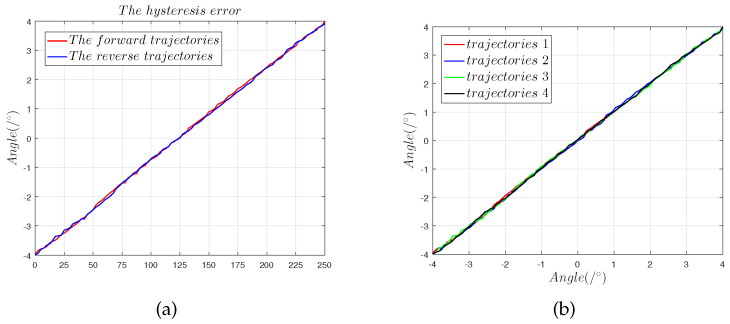
Theoretical and predicted output values of output capacitance for comparison: (**a**) hysteresis error in the 45° direction; (**b**) repeatability error in the 45° direction.

Figure 23 shows that the repeatability error was 0.86%.

#### 4.5.2. Sensitivity and Resolution

The resolution of sensor measurement can be calculated from Equation (16), where the error in the solution is the sensitivity [10]. When the capacitive sensor moves in the 45° direction, $\alpha_{1}$ and $\alpha_{2}$ have the same trend. Taking the output data of the sensor when moving in the 45° direction as an example, the estimated measurement resolution is more representative.

(16) $$\begin{array}{c} R_{es}=\frac{\sigma }{S} \end{array}$$

**Figure 24:**
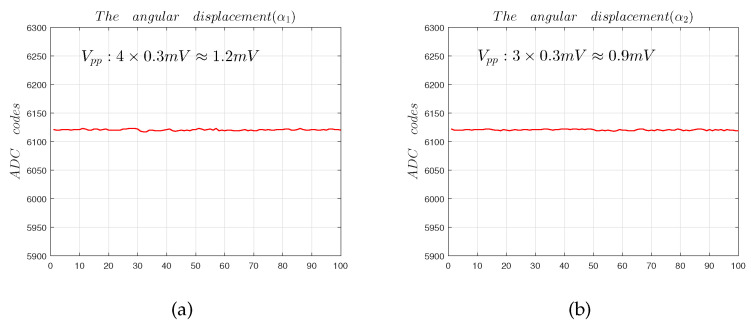
The sensor resolution test: (**a**) angular displacement $\alpha_{1}$ noise calculation; (**b**) angular displacement $\alpha_{2}$ noise calculation.

From Figure 24, we can know that the measurement resolutions of $\alpha_{1}$ and $\alpha_{2}$ are [14]:

(17) $$\begin{array}{c} R_{es1}=\frac{1.2mV}{0.5V{/}^{\circ }}=0.002^{\circ } \end{array}$$

(18) $$\begin{array}{c} R_{es2}=\frac{0.9mV}{0.5V{/}^{\circ }}=0.0015^{\circ } \end{array}$$

#### 4.5.3. Impulse Response

A pulse signal was transmitted to the drive electrode from an external excitation source to test the dynamic performance of the capacitive sensor. The impulse response of the capacitive sensor was simultaneously recorded, and the time constant τ of the capacitive sensor was calculated according to the actual impulse response.

**Figure 25:**
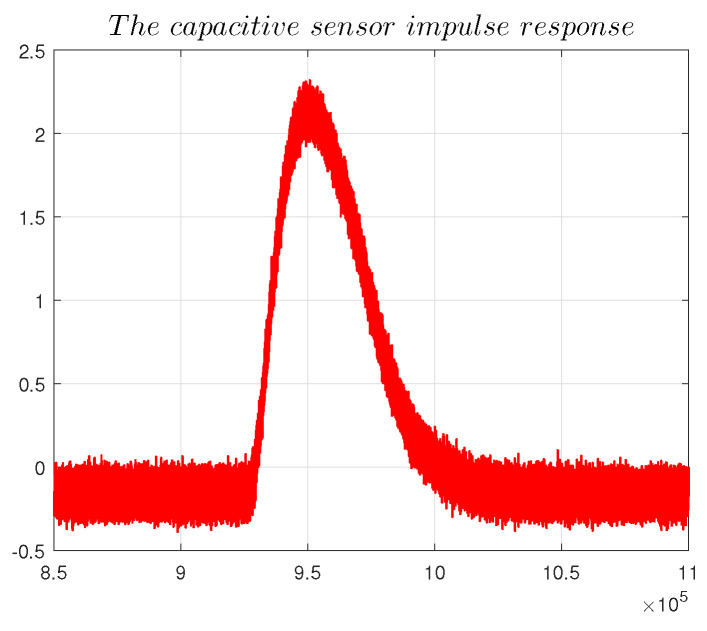
The temperature experiment.

Figure 25 shows that the rise time of the impulse response was 2.246 μs, and the fall time was 6.08 μs.

The capacitive sensor impulse response expression is:

(19) $$y=\left\{\begin{array}{ccc} \frac{kA}{T}\left(1-e^{\left(\frac{-t}{\tau }\right)}\right) & , & 0<t<T,\\ \frac{kA(1-e^{\left(\frac{-t}{\tau }\right)})}{Te^{\left(\frac{-t}{\tau }\right)}}e^{\left(\frac{-t}{\tau }\right)} & , & t>T, \end{array}\right.$$

where *A* is the area of the input pulse, *k* is static sensitivity, and T is pulse signal width. Combining Figure 25 and Equation (19), it can be seen that τ≈ 13 μs, and the settling time *t* of the system is then t=39 μs.

## 5. Conclusions

In this study, we tested the practical application of the designed capacitive sensor in a 2-DOF angle measurement scenario. We first determined the influence of electrode mounting error on the system output during the sensor measurement operation and created a mathematical model of the effects of electrode installation error that we verified using a finite element model. The distribution principle and reduction in installation error were based on the mathematical model and simulation results. We created a prototype experimental platform to detect the 2-DOF angular displacement signals of the capacitive sensor and conducted single-axis and dual-axis measurement experiments. Zero offsets and slope offsets in the measurement data were calculated using the mathematical model, and a compensation process was developed. The capacitive sensor was then tested, and the test results show that the zero offset of the capacitive sensor was reduced to 0.009°, the pulse response time was t=39 μs, and the angle measurement resolution was 0.002° within ±4°.

## Figures and Tables

**Table 1:** Simulation model parameters of the capacitive sensor.

Driving Electrode	Symbol	Value	Unit
The curvature radius	$R_{D}$	127	mm
The projection circle radius	$r_{D}$	60	mm
thickness	$T_{D}$	1	μm
**Sensing Electrode**	**Symbol**	**Value**	**Unit**
Start angle	$\alpha_{start}$	−4	degree (${}^{\circ }$)
Stop angle	$\alpha_{stop}$	4	degree (${}^{\circ }$)
The curvature radius	*R*	126	mm
The projection circle radius	*r*	40	mm
thickness	*T*	1	μm

**Table 2:** Dimensions of the prototype sensor.

Spherical Electrode Dimensional Parameters	Dimension
The curvature radius of the driving electrode ($R_{D}$)	127 mm
The curvature radius of the sensing electrode (*R*)	126 mm
The projection circle radius of the driving electrode ($r_{D}$)	60 mm
The projection circle radius of the sensing electrode (*r*)	40 mm
The gap between the driving electrode and sensing electrode (*d*)	1 mm
